# Infant mortality in South Africa - distribution, associations and policy implications, 2007: an ecological spatial analysis

**DOI:** 10.1186/1476-072X-10-61

**Published:** 2011-11-18

**Authors:** Benn KD Sartorius, Kurt Sartorius, Tobias F Chirwa, Sharon Fonn

**Affiliations:** 1 School of Public Health; Faculty of Health Sciences; University of the Witwatersrand, Johannesburg; South Africa; 2 School of Accountancy; Faculty of Commerce, Law and Management; University of the Witwatersrand, Johannesburg; South Africa

**Keywords:** infant mortality, HIV, spatial analysis, social determinants, attributable fractions, policy implications, Bayesian analysis

## Abstract

**Background:**

Many sub-Saharan countries are confronted with persistently high levels of infant mortality because of the impact of a range of biological and social determinants. In particular, infant mortality has increased in sub-Saharan Africa in recent decades due to the HIV/AIDS epidemic. The geographic distribution of health problems and their relationship to potential risk factors can be invaluable for cost effective intervention planning. The objective of this paper is to determine and map the spatial nature of infant mortality in South Africa at a sub district level in order to inform policy intervention. In particular, the paper identifies and maps high risk clusters of infant mortality, as well as examines the impact of a range of determinants on infant mortality. A Bayesian approach is used to quantify the spatial risk of infant mortality, as well as significant associations (given spatial correlation between neighbouring areas) between infant mortality and a range of determinants. The most attributable determinants in each sub-district are calculated based on a combination of prevalence and model risk factor coefficient estimates. This integrated small area approach can be adapted and applied in other high burden settings to assist intervention planning and targeting.

**Results:**

Infant mortality remains high in South Africa with seemingly little reduction since previous estimates in the early 2000's. Results showed marked geographical differences in infant mortality risk between provinces as well as within provinces as well as significantly higher risk in specific sub-districts and provinces. A number of determinants were found to have a significant adverse influence on infant mortality at the sub-district level. Following multivariable adjustment increasing maternal mortality, antenatal HIV prevalence, previous sibling mortality and male infant gender remained significantly associated with increased infant mortality risk. Of these antenatal HIV sero-prevalence, previous sibling mortality and maternal mortality were found to be the most attributable respectively.

**Conclusions:**

This study demonstrates the usefulness of advanced spatial analysis to both quantify excess infant mortality risk at the lowest administrative unit, as well as the use of Bayesian modelling to quantify determinant significance given spatial correlation. The "novel" integration of determinant prevalence at the sub-district and coefficient estimates to estimate attributable fractions further elucidates the "high impact" factors in particular areas and has considerable potential to be applied in other locations. The usefulness of the paper, therefore, not only suggests where to intervene geographically, but also what specific interventions policy makers should prioritize in order to reduce the infant mortality burden in specific administration areas.

## Background

Despite the Millennium Development Project's aims to reduce infant and child mortality, this problem remains a challenge in sub-Saharan Africa. The infant mortality rate (IMR), moreover, has worsened in many of these countries reversing the gains achieved in the previous century [1][2][3][4][5]. In 1990, for example, there was a 20-fold difference (180 versus 9 deaths per 1000 live births) in IMR between sub-Saharan African and industrialized countries. By 2000, this difference had increased to 29-fold with IMR's of 175 and 6 per 1000 respectively [6] largely as a result of the prevalence of HIV/AIDS in sub-Saharan Africa [7]. Southern Africa, in particular, has been significantly compromised by the HIV/AIDS epidemic both directly through vertical HIV transmission, and indirectly, through maternal death and the absence of a primary care giver [8][9].

Material deprivation is widespread in many sub-Saharan countries. In recent times, the combined effect of material deprivation and HIV/AIDS has negatively impacted on infant mortality. The interactive relationship between HIV/AIDS and material deprivation is illustrated by a combination of increased healthcare costs and a reduced ability to generate income [10]. Furthermore, a wide range of socio-economic variables influence material deprivation including ethnicity, female literacy, maternal mortality and household size. In addition, a lack of social support, unemployment, poor nutrition, access to water, transport and the distance to the nearest healthcare facility are aggravating factors [1][3][9][11][12][13][14][15]. An important variable that measures relative material deprivation in terms of income inequality, namely, the Gini-Coefficient, has also been established as a key determinant of infant mortality [16].

Reducing infant mortality requires a range of investments that include increased health sector spending, improving health systems functioning, and "through socioeconomic progress to improve nutrition, housing, hygiene, education, gender equality, and human rights" [14]. However which investment to make, given resource constraints is not clear. Not only is it not clear which interventions to prioritise, but also whom or where to target the interventions. Reliable statistics on mortality, its causes and trends are in high demand for assessing the global and regional health situation. Reliable mortality data are a prerequisite for planning health interventions, yet such data are often not available in developing countries, particularly in sub-Saharan Africa (SSA). In the absence of such data, alternate data sources need to be utilized to address these gaps and inform progress towards the Millennium Development Goals.

The geographic distribution of health problems such as mortality is also not uniformly distributed and aggregated poverty and health statistics do often not describe the variations in mortality experienced within regions of countries [17][18][19][20]. The IMR, in particular, can vary significantly between geographic locations, as well as across the urban rural divide [21][22]. In South Africa, the incidence of infant mortality differs widely across race groups and provinces [23]. The differential IMR rate is also reflected in unequal socioeconomic status (SES) and access to services and facilities that vary widely across the nine provinces [24].

Population- wide interventions are costly to implement and it is often necessary to target high risk areas [25]. Investigating the distribution and determinants of adverse health outcomes, therefore, can usefully inform more focused and cost effective interventions. In particular, the targeting of high risk health clusters or sub-districts can inform policy planning [1]. Spatial analysis is an important tool in epidemiology to detect possible sources of heterogeneity, spatial incidence or patterns [26]. The potential of spatial analysis is reinforced by the increasing availability of geographically indexed population level data such as mortality, as well as advances in computation methods using GIS systems. Spatial analysis, moreover, can be applied to health data in small area studies [27], as well as to imperfect data, often the case in Africa, through the use of space and time geo-statistics [28].

The objective of this paper is to determine and map the spatial nature of infant mortality in South Africa at a sub district level in order to identify high risk areas and inform policy interventions. In particular, we identify and map high risk clusters of infant mortality, as well as examine the impact of a range of social determinants of infant mortality including maternal health, provincial antenatal HIV sero-prevalence, socio-economic inequality and access to services and facilities at the sub-district level.

In South Africa, little research has focused on spatial differences in mortality at the municipal/sub district level. The identification, targeting and quantification of factors contributing to sub-district level mortality can contribute to more focused public health interventions in South Africa, as well as many other developing countries confronted with similar problems [29]. This paper makes a primary contribution in the health domain by developing a more integrated argument for the determinants of infant mortality in a developing country context [3][4][5]. The paper also makes a contribution by using Bayesian spatial modelling to determine infant mortality at the sub-district or small area level thus extending the conclusions of advanced spatial modelling for public health intervention [30] to interface with service delivery and other indicators. Finally, the paper contributes to the public sector domain by suggesting the use of an infant mortality indicator which can be used as a proxy for the delivery of basic services that influence material deprivation.

## Results

**Figure 1:**
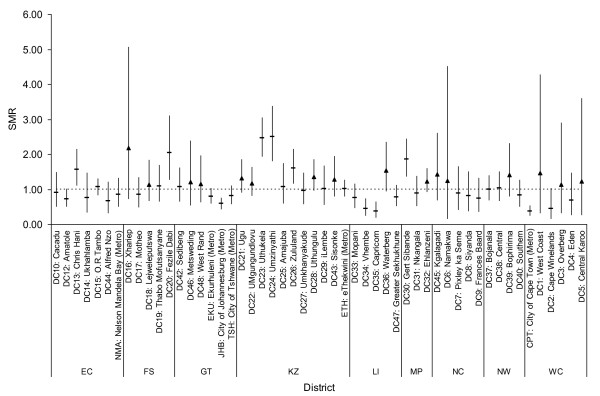
**District level infant mortality rates with 95% confidence intervals and significant high or low districts, South Africa, 2007**. Dashed line represents the national average i.e. SMR = 1; upward triangles represent sub-districts which are not significantly different from SMR = 1 but that are not equivalent based on an equivalency test using a critical SMR range of 0.8-1.25; ISRDP = rural development district; Metro = metropolitan; province abbreviations: EC (Eastern Cape), FS (Free State), GT (Gauteng), KZ (Kwa-Zulu Natal), LI (Limpopo), MPU (Mpumalanga), NW (North West), NC (Northern Cape), WC (Western Cape).

The infant mortality proportions by district (with 95% confidence intervals), illustrated in Figure 1, indicate which districts (n = 53) were significantly above or below the national average (namely a standardized mortality ratio (SMR) of one) using difference tests. It is evident that Kwazulu Natal generally remains the province with the highest infant mortality burden, with 3 of its 11 districts having significantly elevated infant mortality. It is also evident that distinct pockets of excess infant mortality were found in other provinces bordering Kwazulu Natal such as Mpumalanga, Free State and Gauteng. Conversely, all the districts of the Western Cape had significantly lower infant mortality proportions when compared to the overall incidence. We further tried to identify districts which were not significantly different from an SMR of one but that were not equivalent to one, using an equivalency testing approach, as those districts close to a reference value (but not equivalent) are also critical to policy and planning. Those which were not significantly different from one on the positive side (i.e. more than expected infant deaths) but that were not significantly equivalent to one are further highlighted using upward triangles for point estimates in Figure 1. Many of these 'close but not equivalent' sub-districts were identified in Kwazulu Natal (4 districts), already identified as a high risk province based on the difference tests (discussed above).

**Figure 2:**
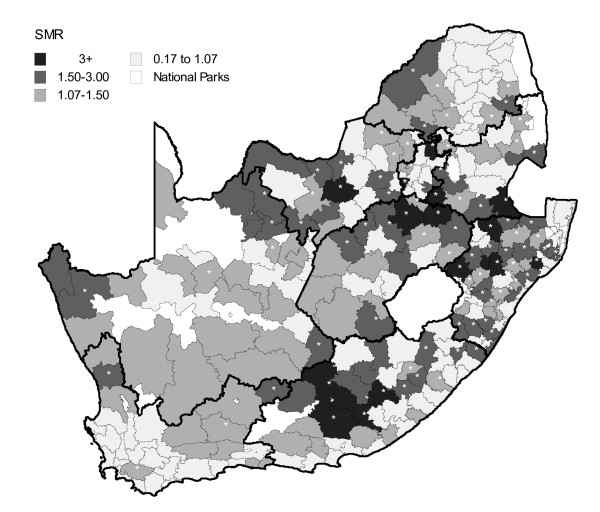
**Bayesian zero-inflated Poisson (ZIP) model (baseline model containing only a constant and the conditional autoregressive parameters - see Appendix 1) showing increasing infant mortality risk by sub-district, South Africa, 2007**. Note: asterisk indicate sub-districts in which the SMR was significantly above 1 based on an exceedance probability of >0.9.

Figure 2 depicts the smoothed standardised mortality ratio (SMR) for infant mortality at the sub-district level (n = 248) using a Bayesian zero-inflated spatial Poisson model. Sub-districts with a SMR significantly above 1 (based on exceedance probabilities from the Bayesian kriging model) are highlighted with asterisk. Areas at lower risk were generally concentrated in sub-districts towards the Western Cape. A band of significant excess infant mortality can be observed stretching across Kwazulu Natal, Mpumalanga and Gauteng with pockets of significant excess mortality in Eastern Cape, Northern Mpumalanga and south-eastern Limpopo (Figure 2).

Indicators were restructured so that increasing level indicates worsening condition. Significant univariate associations between indicators and infant mortality are shown in Table 1. In order to test the multivariable association between infant mortality and its predictors at the sub-district level, various modelling approaches were tested and an augmented Bayesian zero-inflated spatial model proved best. Following multivariable adjustment, maternal mortality had the highest significant adjusted measure of association with infant mortality (IRR = 1.034, p = 0.025). Thus for one unit increase in the proportion scale of maternal mortality we expect a 3.4% increase in infant mortality risk. Antenatal HIV sero-prevalence in 2007 had the next strongest and significant association with infant mortality, followed by increasing ratio of male to female infants (Table 1). Previously sibling mortality was marginally significant following multivariable adjustment (IRR = 1.032, p = 0.088). This is however significantly correlated with antenatal HIV sero-prevalence (ρ = 0.3, p < 0.05) and may partly explain its loss of significance in the final multivariable model. Following multivariate adjustment increasing income inequality and lack of combined service delivery (lack of water, sanitation, refuse and female schooling) were no longer significantly associated with infant mortality.

**Table 1:** Univariate and Bayesian multivariable infant mortality risk factor analysis, South Africa, 2007

Indicator	Univariate analysis		Multivariable analysis	
	**Zero-inflated Poisson model**		**Zero-inflated convolution CAR ^i ^spatial Poisson model**	

	**IRR (95% CI)**	**p-value**	**IRR (95% BCI ^ii^)**	**p-value**

Proportion of previous siblings that have died	1.135 (1.13,1.141)	<0.001	1.032 (0.989,1.081)	0.088

Proportion of mothers that have died	1.108 (1.104,1.113)	<0.001	1.034 (1,1.073)	0.025

HIV antenatal sero-prevalence ^iii ^in 2007	1.035 (1.034,1.037)	<0.001	1.017 (1,1.037)	0.022

Ratio of male to female infants	1.042 (1.041,1.043)	<0.001	1.021 (1.013,1.029)	<0.001

Gini-coefficient for income inequality	1.017 (1.016,1.019)	<0.001	1.003 (0.994,1.014)	0.266

Proportion of females with no schooling	1.031 (1.03,1.032)	<0.001	^v^	---

Poor basic service delivery ^iv^	1.009 (1.009,1.009)	<0.001	^v^	---

Combined lack of female schooling and basic service delivery indicators	1.012 (1.011,1.012)	<0.001	1.003 (0.993,1.002)	0.912

Constant (b0)	---	---	-1.198 (-1.664,-0.727)	---

				

σ^2^_ε _(unstructured sub-district heterogeneity)	---	---	0.446 (0.352,0.552)	---

σ^2^_φ _(spatially structured heterogeneity)	---	---	0.015 (0,0.142)	---

i: Incorporated an unstructured sub-district random effect and a structured normal CAR spatial random effect;ii: Bayesian credibility interval; iii: District level; iv: includes lack of basic service (water, toilet and rubbish disposal) and increasing ratio of infants to clinics within the sub-district

v: in multivariable model we have combined lack of female schooling with poor basic service delivery as they are strongly correlated (ρ = 0.5633, p < 0.01)

Significant high-high spatial autocorrelation (Moran's I) of low service delivery ( for example no refuse disposal, no water service, living more than one kilometre from the nearest water supply), as well as maternal and previous sibling mortality is observed (mostly) in Kwazulu Natal and also in the bordering sub-districts of northern Eastern Cape. High-high spatial autocorrelation of mother death in Northern Mpumalanga and Limpopo is also seen. High-high spatial autocorrelation of households with no income was observed in the North West and Eastern Cape. Conversely, for most indicators a concentration of low-low spatial association can be seen in the Western Cape which had the lowest infant mortality risk.

We also used our final multivariable ZIP model to predict the number of infant deaths for those sub-districts with zero infant deaths sampled (n = 51) based on their indicator profile and estimated a total of 2133 potentially missed infant deaths in this community survey. This potentially revises the total IMR for 2007 from 59.3 to 61.5 per 1000 infant population.

**Figure 3:**
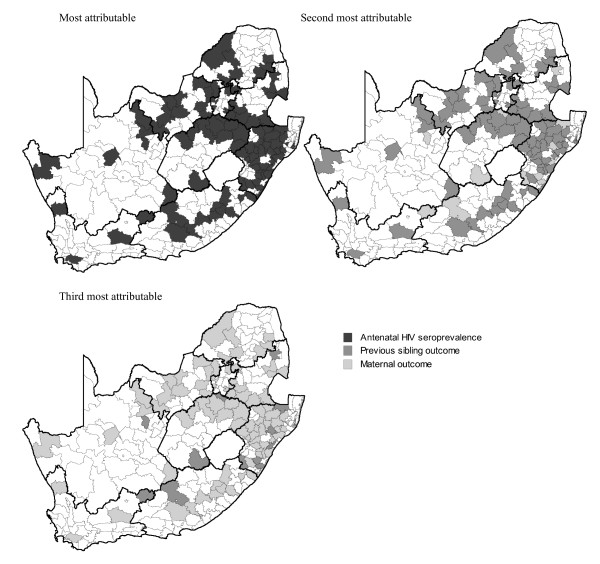
**Risk indicators with highest attributable fractions (impact) in significantly high risk infant mortality sub-districts, South Africa, 2007**.

Furthermore, we combined the degree of association of each predictor with its sub-district level prevalence (attributable fraction) in high infant mortality sub-districts (n = 98) to assess the relative importance of the predictors in a given sub-district. In other words a predictor may have a strong association with infant mortality but have a low sub-district level prevalence and thus not adequately explain the burden of infant mortality in that sub-district from a public health or policy point of view. Figure 3 displays the most attributable predictors (highest impact) in the high risk infant mortality sub-districts. We found that antenatal HIV sero-prevalence in 2007 appears to have the largest attributable impact (98 of 98 high risk sub-districts). At a secondary level (attributable risk) the proportion of lastborn that died appears to have had the highest impact (86/98) followed by proportion of maternal deaths (12/98). For the tertiary attributable estimates maternal mortality was the most attributable indicator (86/94) followed by previous sibling mortality (12/94). Finally, primary level attributes (HIV sero-prevalence) accounted for 34% of infant mortality on average in the high risk sub-districts, followed by 14% for secondary (previous sibling mortality; maternal mortality) and 4% for tertiary (maternal mortality; previous sibling mortality).

## Discussion

The results confirm that infant mortality remains a problem in South Africa and that it has been escalated by the impact of the HIV/AIDS epidemic, confirming similar findings in sub-Saharan Africa that are contrary to improving trends in most other parts of the world [3][5][31][32]. Previous research by Nannan et al [33] showed a rise in infant mortality in South Africa from 39 per 1000 in 1992 to 56 per 1000 in 1997. Garrib et al. [8] also estimated the infant mortality ratio over the period 2000 to 2002 at 59.6 per 1000 live births. The HIV epidemic has had a pronounced impact on infant mortality in South Africa because it effects infant survival directly (mother to child transmission), as well as indirectly as a result of maternal (and paternal) mortality and the loss of working adults [9][10][34]. The results estimate an infant mortality rate of 59 per 1000 in 2007 thus confirming that the levels observed in the late 1990's and early 2000's have seemingly not reduced by 2007. This is despite national anti-retroviral therapy (ART) rollout starting in 2004 which should presumably improve both maternal survival and strengthen prevention of mother-to-child transmission programmes (PMTCT) using highly active anti-retroviral therapy (HAART). Furthermore, the results show that the risk of infant mortality is not uniformly distributed and a band of significant excess infant mortality is observed stretching from the Eastern Cape, through Kwazulu Natal and north into parts of Mpumalanga and Gauteng. In particular, infant mortality in rural areas was high and lower mortality was observed in the 6 metropolitans (district level equivalent area). Conversely significantly lower infant mortality was observed in most of the Western Cape thus confirming the findings of earlier studies in South Africa [23].

Contrary to other sub-Saharan countries, South Africa's high infant mortality rate has occurred against a backdrop of sustained economic growth between 1994 and early 2010. Economic growth (GDP), however, differs markedly across its nine provinces and the results show high levels of income inequality (Gini-Coefficient) thus confirming the earlier findings of Booysen et al. [35]. As with other developing countries [17][18][20] programs are more readily accessed by wealthier households. Poorer households are excluded because of comparatively higher direct and indirect costs to access facilities [36], especially in an environment of rising costs [37]. Furthermore, the development of the national economy has, in most instances, been centred around the major urban metropolis at the expense of rural South Africa where 70 per cent of South Africa's poor live [38,39]. Rural South Africa, therefore, not only experiences high levels of poverty and income inequality, but also less access to services and facilities. These facilities include infrastructure, clinics, water and sanitation that vary widely across the rural urban divide, as well as across the nine provinces. The risk of infant mortality, therefore, appears to be increased by a material deprivation squeeze that combines income inequality on the one hand with the unequal distribution of disease and other services. The results reflect the findings in a number of other developing countries [12].

The results suggest a complex and highly correlated array of determinants that influence infant mortality in South Africa. As mentioned maternal death emerged as a prominent risk factor for infant mortality in this study and has been shown previously [9]. A higher proportion of male than female infants within a given sub-district remained a risk following multivariable adjustment. Explanations for this gender difference in infant mortality are dominated by biological factors [40] and form part of core analytical frameworks for assessing infant survival [41]. The death of the previous sibling as a predictor of current infant outcome has been shown previously [42,43] and might suggest that the current infant would have had a survival disadvantage given certain unfavourable endogenous and exogenous factors (i.e. high risk women, families or households) faced by the previous sibling. Women who have experienced prior child deaths should be given special care in prenatal clinics and these sub-districts should be targeted [43]. Given these complexities, however, the direct and indirect impacts of HIV are evident and improving prevention of vertical transmission of HIV, as well as ensuring maternal survival in identified high risk sub-districts, is key to reducing infant mortality in these hotspots. Strategies include the prevention of HIV infection, expanded antenatal testing, prevention of mother-to-child transmission, improved access to ART and correct breastfeeding practices [9]. Increasing proportion of females within the sub-district with no schooling was found to be a risk based on the univariate analysis. This conforms with similar findings between sub-district education attainment (in this case female) and infant (and child) survival [44,45], as well as underlining the need for multiple parallel interventions. In this regard, education has a direct impact on health related behaviour and choices [4] and educated households are more likely to access prenatal care and health services [46], as well as adopt better hygiene practices [45,47]. Of those with no schooling, the vast majority were African that had a significantly higher proportion of mothers and fathers that were dead. A lack of schooling was also significantly linked to the higher fertility (parity) or number of children, as well as a higher unemployment proportion. In parallel to HIV programme, interventions should be accompanied by programs to relive material deprivation because a lack of basic services (like power, water and refuse removal), improve education and potentially poor access to health care was found to be significantly linked to the increased risk of infant mortality [3,5,23].

The results thus confirm the complex and relative ecological contribution of various social and biological determinants on infant mortality.

## Conclusions

This paper has demonstrated the potential of advanced spatial techniques and Bayesian modelling to identify administrative areas at sub-district level with significantly higher infant mortality than the national norm. The paper highlights the multitude of key social and biological determinants of infant mortality using a multivariable Bayesian modelling approach to adjust for inherent correlation between neighbouring sub-districts. Combining determinant prevalence at the sub-district level with multivariable risk factor estimates has not been widely used to identify the most attributable factors. This more integrated estimate will assist policy makers in countries with high infant and child mortality burden.

The usefulness of these results is that they investigate a more complex interplay of variables that explains why, even in areas where there is economic growth, infant mortality can still be an intractable problem because of high levels of income inequality. The use of Bayesian hierarchical modelling also best deals with the problems posed by small area studies such as absent data as discussed earlier. The usefulness of these results is further enhanced by the use of advanced GIS mapping of infant mortality risk and the associated predictor distributions at the smallest administrative unit. These spatial maps are able to pinpoint areas within a country with higher levels of mortality, as well as explain the most likely reasons for this persistent problem based on attributable fractions. This study underscores the need for exploratory and advanced approaches to assess within-country geographic patterns of all-cause infant mortality using national sub-district level ecological data.

Addressing the infant mortality in South Africa requires a multifaceted and targeted approach. The results can be potentially useful to government planners for policy intervention purposes within specific sub-districts. What is also clear is that policy development will need to coordinate a wide range of government departments in order to reduce infant mortality. These departments or sectors include planning and coordination, health, education, finance, public works and industry because of the wide range of social determinants. Finally, the results are useful because infant mortality and its social determinants can be a useful proxy to monitor the efficiency of service delivery at sub-district level.

This study is limited by the data that were included in the community survey. Thus, variables not included may have a significant impact on our model. The consistency of our findings with international trends would suggest that our model is reliable and based on the same kind of data. Given the ecological (aggregated) nature of the data caution should therefore be taken with interpretation of the direct causal inferences found in our multivariable analysis. The data were extracted down to the smallest administrative areal unit available (namely local municipality or sub-district) which we believe reduces the ecological effect in part. Sampling error may also affect our findings, however, we did use the weights provided by Statistics South Africa which would in part compensate for this potential bias.

Current government commitments in South Africa suggest increasing and improving HIV services, as well as improved access to education and service delivery. Given these changes, as well as the limitations of our paper, we propose that this study is replicated using future Statistics South Africa data, as well as in other African settings.

## Methods

### The study area

**Figure 4:**
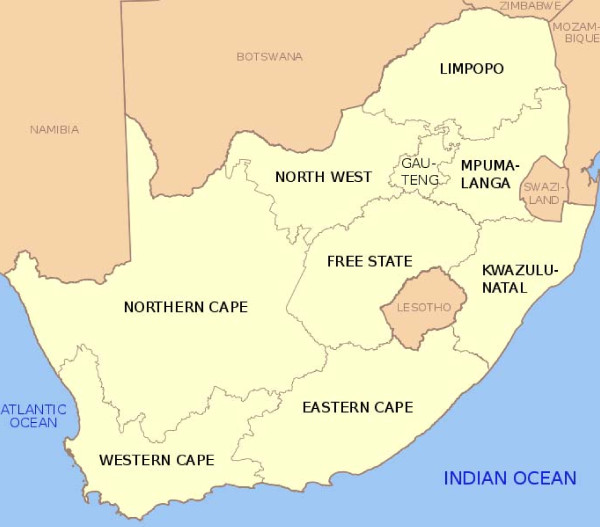
**Map of South Africa, with provinces and neighbouring countries**.

South Africa is administratively divided into nine provinces responsible for health service delivery (Figure 4), and further divided into 53 districts [47 district municipalities and 6 metropolitan districts. These districts are then disaggregated into 248 local municipalities. Service delivery for water and sanitation is a municipal function in most instances.

The nine provinces vary in a number of ways. The Western Cape has the highest human development index (HDI) ^1 ^followed by Gauteng [48], while the ten most deprived districts in 2007/2008 were located in Kwazulu Natal (6), Eastern Cape (3) and Limpopo (1) that are all classified as rural development districts [49]. Conversely all the districts within the Western Cape were classified as the least deprived as were three of the six metros, namely the City of Cape Town and the Nelson Mandela metro (Eastern Cape) and the City of Johannesburg (Gauteng).

### The data

The data were drawn from the community survey run by Statistics South Africa in 2007. These data included information regarding demographic indicators (fertility, mortality and migration) and socio-economic data that included poverty indicators, access to facilities and services and levels of unemployment [50]. The 2007 Community Survey randomly sampled enumeration areas (EA) and then dwelling units within each EA. An enumeration area is defined as the smallest geographical unit (piece of land) into which the country is divided for enumeration purposes. Enumeration areas contain between 100 to 250 households. The survey indicted 80,787 EA's countrywide and 1,321 were excluded as they were designated as institutions or recreational areas. The EA's within each municipality were ordered by land use and human settlement type and selection was done using systematic random sampling. The second level of the sampling frame consisted of re-listing the dwelling units (which could potentially contain one or more households) within the selected EA's. Random selection of dwelling units was based on a fixed proportion of 10% of the total listed dwellings in an EA. The survey sample covered 274 348 dwelling units across all the provinces and attained a response rate of 93.9% [51]. In this regard, the recalculation of person weights to address sampling errors was applied to provide more credible estimates of the population at national and provincial levels. Data based on these weights were used in the analysis in this paper.

The South African Statistics Council [52] found the reported demographic data (fertility and mortality proportions) to be entirely plausible when compared to other censuses. Certain limitations and potential errors were identified by Statistics SA and the South African Statistical Council when reviewing the survey. The following systematic errors were observed in the data:

-Underestimate of men relative to women;

-Underestimate of children younger than 10 years;

-Excess of people aged 10-24 in Western Cape and Gauteng; and

-Deficit of women aged 20-34 in Free State, KwaZulu-Natal and Limpopo.

The following aggregated (ecological) sub-district level data were extracted (Nesstar) from the primary Community Survey 2007 database: infant population and deaths; maternal (deaths, fertility, and if a previous sibling(s) to the current infant had died); paternal (deaths); sub-district education level, employment status and household income; household services (access to water, water type and distance to nearest water source; household toilet facilities; household refuse removal). We also calculated Gini-coefficient, a commonly used measure of inequality, for each of the sub-districts based on the dispersion of annual household income within that sub-district. Additional data regarding district level antenatal HIV sero-prevalence in 2007 were extracted from the District Health Barometer for 2007/2008 [49]. Other data sources: additional district level data on HIV antenatal seroprevalence and the number of clinics in each distract are taken from District Health Barometer for 2007/2008 [49].

Finally, a national shape file containing all 248 sub-districts was imported into MapInfo Professional 9.5 to create the necessary areal and geospatial data. Centroids of each sub-district, as well as an adjacency matrix of all neighbouring sub-district combinations were extracted using functions within this software package. These centroids and the adjacency matrix were needed for the various spatial and multivariable analyses (autocorrelation, clustering and Bayesian conditional autoregressive approaches) described in detail below.

### District infant mortality proportions

The infant mortality proportions were calculated for each district by dividing the observed number of deaths by the total population in district i (i = 1,...,52) based on the weighted 2007 community survey. To identify districts in which the mortality proportion was significantly above average, we constructed the exact 95% confidence intervals for each rate using the Poisson distribution of the observed number of deaths [53]. District mortality was considered significantly above average for that year if the overall proportion for the given year was below the lower limit (α = 0.025) of the mortality proportion for that district [54]. This approach does not allow conclusions for districts close to the reference value (SMR = 1) which are equally crucial to policy makers. The combined approach of difference and equivalence testing has recently emerged as a way to improve the interpretability of areal spatial data [55]. Thus for districts which were not significantly different from the reference value but that were greater than 1 (SMR>1) we also performed equivalency testing using a typically used critical value of Δ = 0.2 [56], which leads to an equivalence range of (0.8, 1.25). We used the twice-the-smaller-tail (TST) method [57] which is an computation of the equivalence test statistic for discrete distributions (i.e. Poisson in this case).

### Spatial analysis

Various spatial analysis techniques and models were employed in this study to compare and identify significant infant mortality "hotspots", namely Moran's I spatial autocorrelation coefficient [58], Kulldorff spatial scan statistic [59], a standard Bayesian convolution conditional autoregressive approach [60] and lastly a Bayesian augmented zero-inflated Poisson approach [61,62]. The first three each have inherent strengths and weaknesses which are extensively detailed in the literature. Further detail regarding the Bayesian approaches are provided in this paper. Given the similarity of the output for infant mortality risk from these various approaches, we only present results for the final Bayesian approach (see Appendix 1 for details of model assessment). We did however use Moran's I to test both for significance of values within a sub-district as well as a measure of the strength of clustering or dispersion of the various indicator variables [63]. Exceedance probabilities (i.e. smoothed standardised mortality ratio in given area significantly greater than 1) from the Bayesian spatial modelling approach were used to identify sub-districts with significant excess infant mortality risk in the attributable fraction analysis. This is further detailed in Appendix 1.

### Bayesian spatial modelling of sub-district infant mortality

In order to address the problems associated with small area analysis and spatial correlation, we finally used Bayesian hierarchical modelling. Small area studies have better interpretability than larger scale studies and are less susceptible to ecological fallacy or bias. However the drawbacks include data that may be very sparse with a large number of event free (zero count) area and over-dispersion of the data [26]. Correlation or interdependence of observations in neighbouring or adjoining areas also poses a problem. Objects (in this case sub-districts) in close proximity are often more alike. Consequently, it is important to include the effects of spatial proximity when performing statistical inference on such processes. The standard error of the covariates, moreover, is underestimated if this spatial correlation is not taken into account, thereby overestimating the significance of the risk factors. The estimates of the outcomes, such as mortality, are also incorrect at the locations where data are missing. Bayesian areal or geostatistical models relax the assumption of independence and assume that spatial correlation is a function of neighbouring locations or distance between locations and also allows prediction at unsampled locations [64]. Lastly, measurement errors for both numerators and denominators also represent a problem associated with small area studies [27]. Bayesian hierarchical models are the most commonly used framework to address the problems posed by small area analysis [65]. Bayesian estimators are also widely used in order to obtain reliable estimates for the relative risk when there are sub-areas with small populations and traditional estimates of relative risk lead to unreliable or unstable results [66].

With the development of Markov Chain Monte Carlo (MCMC) methods and software such as OpenBUGS, Bayesian approaches are being increasingly applied to the analysis of many social and health problems in addition to disease mapping and modelling. Two different Bayesian spatial model formulations were tested and used in this study. These models were based on fitting spatial Poisson models with two random-effects terms that took the following into account: (1) sub-district contiguity [spatial term); and (2) sub-district heterogeneity. We firstly used the Besag, York and Molliè [60] or convolution conditional autoregressive (CAR) model that is discussed in more technical detail in Appendix 1. For the spatial risk map we used a formulation of the above which included no covariates (only a constant and the convolution conditional autoregressive terms).

### Univariate and Bayesian multivariable risk factor analysis

To calculate expected outcomes (E_i_), the overall infant mortality for 2007 was multiplied by each sub-districts infant population to give the expected number of infant deaths. The following indicator variables were tested against infant mortality: maternal mortality; previous sibling(s) outcome; education, household income and Gini-coefficient derived from income; household services (access to water, household toilet facilities; household refuse removal; ratio of infants to sub-district clinics).

In order to assess the relationship between infant mortality and the various predictors, preliminary univariate zero inflated Poisson regressions were run in Stata 10.0 SE. Covariates significant at the 10% level were then incorporated into the multivariable Bayesian spatial model. Details of the multivariable model are provided in Appendix 2.

### Attributable fractions

We wanted to assess the degree to which sub-district exposure to a particular variable (e.g. access to water and sanitation) impacted on infant mortality. This could provide an indication for policy makers about what intervention(s) to prioritise. To do this we linked together the risk estimates associated with the indicators in the multivariable model with the actual prevalence of exposure to those indicators within the various high risk sub-districts identified through our spatial analysis. The following standard formula for calculating an attributable fraction (AF) for each determinant based on its prevalence of exposure (*p*_e_) in a given sub district, as well as the model coefficient (IRR) for that determinant was used:

Finally, the analysis was carried out in STATA 10.0 SE, SaTScan and OpenBUGS. Maps were developed in Stata 10.0 SE and MapInfo Professional 9.5.

## List of abbreviations

AF: Attributable Fraction; AIDS: Acquired Immunodeficiency Syndrome; ART: Anti-Retroviral Therapy; CAR: Conditional Autoregressive; EA: Enumeration Areas; GDP: Gross Domestic Product; HAART: Highly Active Anti-Retroviral Therapy; HDI: Human Development Index; HIV: Human Immunodeficiency Virus; IMR: Infant Mortality Rate; IRR: Incidence Risk Ratio; MCMC: Markov Chain Monte Carlo; PMTCT: Prevention of Mother-To-Child Transmission; RR: Relative Risk; SMR: Standardised Mortality Ratio.

## Competing interests

The authors declare that they have no competing interests.

## Appendix 1: Bayesian Spatial Modelling

The Besag, York and Molliè [60] or convolution conditional autoregressive (CAR) model is the most widely used spatial Poisson model (for lattice or areal data) in epidemiology [for example: [26]) and is formulated as follows:

$${\text{O}}_{\text{i}}\sim \text{Poisson}\left({\text{E}}_{\text{i}}\lambda_{\text{i}}\right)$$

$$\log\left(\lambda_{\text{i}}\right)=\alpha +\varepsilon_{\text{i}}+\varphi_{\text{i}}$$

where λi is the relative risk in area i, O_i _is the number of infant deaths in sub-district i, E_i _are the expected deaths, ε_i _is the sub-district heterogeneity term and φ_i _is the conditional autoregressive (CAR) spatial term. The spatially correlated random effect of the ith region (φ_i_) is based on the sum of the weighted neighbourhood values. We used an adjacency matrix of common boundaries of a given sub-district to model this spatial association. The unstructured sub-district level random effect was modelled as independent normal distribution ε_i _~ N (0,σ^2^_ε_) with variance σ^2^_ε_. Besag et al argue that this convolution model is more flexible than assuming a CAR random effect only, since it allows the data to decide how much of the residual disease risk is due to spatially structured variation, and how much is unstructured over-dispersion [60]. Specifications for these parameters are given below in the risk factor analysis section. The Besag, York and Molliè (BYM) model does have certain limitations. It is common to find excess zeros in many count data greater than that expected by a Poisson distribution. As a result the observed counts do not follow a Poisson which can lead to inconsistent estimates.

Secondly, we used a zero-inflated Poisson (ZIP) model as the data displayed over dispersion and this modelling allows for the inclusion of a large number of event free areas [67,68]. These models are constructed as a mixture of a Bernoulli and Poisson distribution and contain a mixture point mass at zero and an untruncated count distribution. We used an augmented ZIP model proposed by Rodrigues [61] and Ghosh et al. [62]. It is formulated as follows:

$${\text{O}}_{\text{i}}\sim \text{Poisson}\left(\lambda_{\text{i}}\right)$$

$$\lambda_{\text{i}}={\text{I}}_{\text{i}}*\mu_{\text{i}}$$

$$\log\left(\mu_{\text{i}}\right)=\log\left({\text{E}}_{\text{i}}\right)+\alpha +\varepsilon_{\text{i}}+\varphi_{\text{i}}$$

$${\text{I}}_{\text{i}}\sim \text{Bernouli}\left(\text{p}\right)$$

$$\text{p}\sim \text{beta}\left(\text{1},\text{1}\right)$$

where I is the indicator to distinguish between excess zero counts and non-excess zero or non-zero counts (I = 0 for excess zero counts and I = 1 for non-zero counts) and p is the probability of non-excess zero counts [62]. The above two formulations were adopted as the basic kriging models which include no covariates.

The Deviance Information Criterion (DIC) [69] was used as the criterion for assessment the goodness of fit and thus model selection. Based on the deviance information criterion (DIC), the lower the DIC the better the model fit, the zero-inflated spatial Poisson model performed far better than the BYM spatial Poisson model. Thus results for the ZIP model are presented both for sub-district relative risk estimation and for the multivariable predictor modelling discussed below. The Vuong test of a zero-inflated Poisson versus a standard Poisson, run in Stata was also highly significant (p < 0.001) indicating that the zero-inflated model was better.

The results of the ZIP model were plotted on sub-district maps that depicted smoothed standardised mortality ratio (SMR) estimates and the distribution of the posterior probability that SMR<1 or >1. We used a modified (more stringent) version of Richardson's criterion [70], in which probabilities in excess of 0.9 (Richardson's standard criterion is 0.8) were deemed to be significant.

## Appendix 2: Multivariable risk factor analysis

The Bayesian multivariable augmented ZIP model were simply an extension of the kriging model discussed above with the inclusion of covariates:

$$\log(\mu_{\text{i}})=\log({\text{E}}_{\text{i}})+\alpha +\varepsilon_{\text{i}}+\varphi_{\text{i}}+\text{Xi}\beta$$

where Xi are the vector of covariates and β is the vector of regression coefficients. We used μ_i _to estimate the expected number of missed deaths for zero count sub-districts.

The models were fitted using Markov chain Monte Carlo simulation methods with non-informative priors [65]. Vague Normal distributions were used for β, and inverse gamma priors for the variance parameters. Coefficients for indicators were exponentiated to represent incidence risk ratios (IRR). Posterior distributions of parameters were obtained using WinBUGS [71]. MCMC simulation was applied to fit the models. A two-chain Markov chain Monte Carlo simulation was used for parameter estimation. Model convergence was assessed by visual inspection of the series plot of each parameter, and using Gelman-Rubin statistics [72]. The final posterior samples obtained after convergence were run until the Monte Carlo error for each parameter was less than 5% of the sample standard deviation. We also assessed convergence by running two separate chains. The chains were then sampled until a sample size of 10,000 iterations had been attained.

## Authors' contributions

BKDS contributed to the conception and design, analysis, interpretation of results, drafted manuscript. KS contributed to interpretation of results, assisted with drafting the manuscript. TFC contributed to interpretation of results and reviewed manuscript. SF contributed to interpretation of results and reviewed manuscript. All authors read and approved the final manuscript.

## Endnotes

^1^Composite measure of life expectancy, literacy, education and standard of living and is used globally to rank countries.
